# The mineralocorticoid receptor leads to increased expression of EGFR and T-type calcium channels that support HL-1 cell hypertrophy

**DOI:** 10.1038/s41598-021-92284-y

**Published:** 2021-06-24

**Authors:** Katharina Stroedecke, Sandra Meinel, Fritz Markwardt, Udo Kloeckner, Nicole Straetz, Katja Quarch, Barbara Schreier, Michael Kopf, Michael Gekle, Claudia Grossmann

**Affiliations:** Julius Bernstein Institute of Physiology, Martin Luther University Halle-Wittenberg, Magdeburger Str. 6, 06097 Halle, Saale, Germany

**Keywords:** Physiology, Diseases

## Abstract

The EGF receptor (EGFR) has been extensively studied in tumor biology and recently a role in cardiovascular pathophysiology was suggested. The mineralocorticoid receptor (MR) is an important effector of the renin–angiotensin–aldosterone-system and elicits pathophysiological effects in the cardiovascular system; however, the underlying molecular mechanisms are unclear. Our aim was to investigate the importance of EGFR for MR-mediated cardiovascular pathophysiology because MR is known to induce EGFR expression. We identified a SNP within the EGFR promoter that modulates MR-induced EGFR expression. In RNA-sequencing and qPCR experiments in heart tissue of EGFR KO and WT mice, changes in EGFR abundance led to differential expression of cardiac ion channels, especially of the T-type calcium channel CACNA1H. Accordingly, CACNA1H expression was increased in WT mice after in vivo MR activation by aldosterone but not in respective EGFR KO mice. Aldosterone- and EGF-responsiveness of CACNA1H expression was confirmed in HL-1 cells by Western blot and by measuring peak current density of T-type calcium channels. Aldosterone-induced CACNA1H protein expression could be abrogated by the EGFR inhibitor AG1478. Furthermore, inhibition of T-type calcium channels with mibefradil or ML218 reduced diameter, volume and BNP levels in HL-1 cells. In conclusion the MR regulates EGFR and CACNA1H expression, which has an effect on HL-1 cell diameter, and the extent of this regulation seems to depend on the SNP-216 (G/T) genotype. This suggests that the EGFR may be an intermediate for MR-mediated cardiovascular changes and that SNP analysis can help identify subgroups of patients that will benefit most from MR antagonists.

## Introduction

The mineralocorticoid receptor (MR) is a steroid hormone receptor that is part of the renin–angiotensin–aldosterone system (RAAS). It regulates water-electrolyte homeostasis and contributes to long-term blood pressure control. Independently of its effect on blood pressure, the MR can also directly mediate pathophysiological changes in the cardiovascular system. The MR has been extensively studied in cardiomyocytes, vascular smooth muscle and endothelial cells as well as in fibroblasts and immune cells. Large clinical trials have shown that treatment with MR antagonists leads to a substantial reduction in mortality and morbidity in patients with heart failure of different etiologies^1,2^. As underlying mechanism, a reduction in structural and electrical remodeling of the heart and vasculature has been discussed without the exact molecular mechanisms being clear. In different cell and animal models, activated MR has been shown to play a role in cardiovascular hypertrophy, fibrosis, remodeling, inflammation and endothelial dysfunction and arrhythmias^3–11^. Activation of the MR physiologically occurs through binding of its ligand aldosterone, which induces cytosolic-nuclear shuttling and binding to hormone-response-elements called GREs (glucocorticoid response elements), which it shares with its close relative, the glucocorticoid receptor. Only few MR-specifically regulated genes with pathophysiological relevance for the heart are known but would be candidates for prevention and therapy of cardiovascular disorders. As one candidate gene, we identified the epidermal growth factor receptor (EGFR), a receptor tyrosine kinase, as a specifically MR-regulated gene.

The epidermal growth factor receptor (EGFR) is a ubiquitously expressed membrane receptor tyrosine kinase known to induce cell growth and differentiation and to play a role in cancer biology^12^. Furthermore, a role in cardiovascular physiological and pathophysiological processes has been recognized and an expression in vascular cells (endothelium and smooth muscle cells), cardiomyocytes, fibroblasts and immune cells (macrophages, monocytes, plasma cells, T-cell, neutrophils) has been demonstrated. Functionally, the EGFR is involved in cell proliferation, cell survival, migration, hypertrophy and remodeling. Accordingly, blocking the EGFR seems to be cardioprotective^13,14^. Ligands of the EGFR include EGF, transforming growth factor-α, heparin-binding EGF-like growth factor (HB-EGF), amphiregulin, betacellulin, epiregulin, and epigen. They are transmembrane precursors and are cleaved by matrix metalloproteases. All ligands have been reported to be expressed in the heart and vasculature and for most ligands an involvement in cardiovascular pathologies has been shown. For example, HB-EGF plays a role in heart development and maintenance of cardiac function^15–17^, amphiregulin aggrevates cardiac fibrosis and dysfunction after myocardial infarction and is a potent mitogen for vascular smooth muscle cells (VSMCs)^18–20^, betacellulin enhances DNA synthesis and angiogenesis^20,21^ and is located in atherosclerotic plaques of human aorta^22^ and epiregulin is a potent vascular smooth muscle cell-derived mitogen induced by angiotensin II, endothelin-1, and thrombin^23^. Transforming growth factor-alpha mediates nuclear factor kappa B activation in strained arteries^24^. In addition the EGFR can be transactivated by G protein-coupled receptors of well-known cardiovascular mediators like angiotensin-II, phenylephrine or endothelin-1, which exert part of their pathophysiological effects through this mechanism^25^.

In previous studies, we demonstrated transcriptional regulation of the EGFR by the MR and characterized an MR-responsive element, where MR binds and SP1 functions as a cofactor^26,27^. The effect was MR-specific and not glucocorticoid receptor-inducible and therefore we hypothesized that part of the pathological MR actions are mediated by this pathway. We now found that there is a SNP (-216 G/T) within the SP1 binding site of the EGFR promoter that largely affects the magnitude of the MR-mediated response on EGFR promoter activity. This indicates that a subgroup of the population is especially prone to changes in EGFR expression and possibly also to MR-mediated cardiovascular diseases. To find a correlation between MR, EGFR and cardiovascular disorders, we explore the effect of changes in EGFR expression on cardiovascular gene expression. In WT compared to EGFR KO mice, we found an overrepresentation of differentially regulated ion channels in heart tissue. Further analyses revealed that T-type calcium channels were aldosterone and EGFR-dependently regulated in heart tissue and cardiomyocytes of EGFR WT and KO animals. In HL-1 cells, aldosterone- and EGF-dependent changes in peak current density of T-type calcium channels were measured and an accordant increase in CACNA1H protein expression was confirmed by Western blot. No changes in activation or inactivation of I_CaT_ was found. EGFR inhibition abolished the effect of aldosterone on CACNA1H protein expression. Prelimary results in neonatal rat cardiomyocytes supported our protein expression data from HL-1 cells. Pharmacological inhibition of T-type calcium channels reduced HL1 cell diameter, volume and BNP levels, indicating that CACNA1H is a potential mediator for cardiovascular changes. Overall, the role of EGFR as part of a specific MR signaling pathway is characterized and its possible relevance for pathological MR effects in the heart is explored.

## Results

### MR, SP1 and different EGFR promoter variants

**Figure 1:**
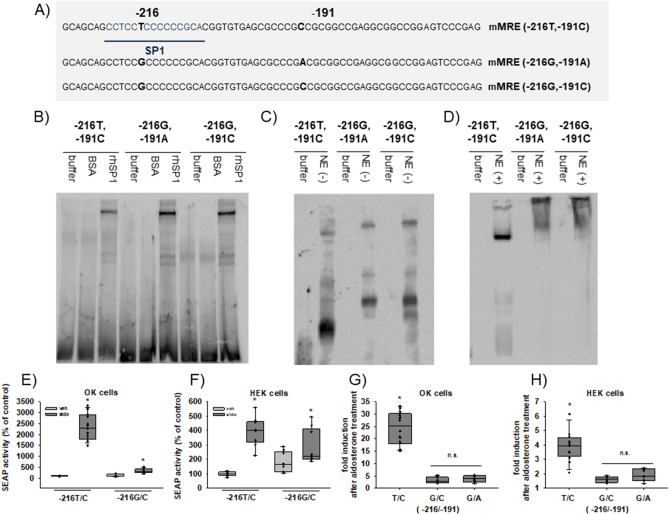
Effect of SNPs on MR-dependent EGFR expression and SP1 binding. (**A**) Sequences of the different mMRE variants and the SP1 consensus sequence (SP1) are depicted. (**B**) EMSAs were performed with biotinylated mMRE (-216T/-191C), mMRE (-216G/-191A) or mMRE (-216G/-191C) probes as indicated. Probes were either incubated with buffer, 300 ng BSA as control for unspecific protein interactions or with 300 ng rhSP1 (representative EMSA of N = 4). (**C**) EMSAs were performed with biotinylated mMRE (-216T/-191C), mMRE (-216G/-191A) or mMRE (-216G/-191C) probes as indicated. Probes were incubated with nuclear extracts from unstimulated hMR-transfected HEK cells (NE (−)) or buffer (representative EMSA of N = 4). (**D**) EMSAs were performed with biotinylated mMRE (-216T/-191C), mMRE (-216G/-191A) or mMRE (-216G/-191C) probes as indicated. Probes were incubated with nuclear extracts from hMR-transfected aldosterone-stimulated HEK cells (NE (+)) or buffer (representative EMSA of N = 4). (**E**) OK cells transfected with hMR and mMRE-216 (G/T) variants coupled to a SEAP reporter gene were incubated for 48 h with either vehicle or 10 nM aldosterone (N = 3, n = 6–9). (**F**) HEK cells transfected with hMR and mMRE-216 (G/T) variants coupled to a SEAP reporter gene were incubated for 48 h with either vehicle or 10 nM aldosterone (N = 3, n = 6–9). (**G** and **H**) Fold induction achieved by aldosterone in OK or HEK cells transfected with hMR and mMRE variants.

In previous studies we showed that binding of activated MR and specificity protein 1 (SP1) to a 65 bp long fragment of the EGFR promoter induces EGFR expression^27^. When characterizing the novel minimal MR responsive element (mMRE) of the EGFR promoter, we found that two single nucleotide polymorphisms (SNPs) are located within the mMRE and that one SNP is located in the predicted SP1 binding site. Therefore, we analyzed the interaction of SP1 with the MRE variants by performing electromobility shift assays with biotinylated probes and either recombinant human SP1 (rhSP1) or SP1 from nuclear extracts of MR-transfected HEK cells incubated with aldosterone or vehicle. Sequences of the mMRE SNP probes as well as of the characterized SP1 binding site are depicted in Fig. 1A. All mMRE variants showed a retention band at an identical position in the gel after incubation with rhSP1 although the band was less prominent for mMRE (-216T,-191C) compared to mMRE (-216G,-191C). SNP-191 had no effect on rhSP1 binding when comparing mMRE (-216G,-191A) and mMRE (-216G,-191C). Incubation with bovine serum albumin as negative control for non-specific protein binding yielded no retention band (Fig. 1B). Incubation of the different probes with nuclear extracts of MR-transfected HEK cells without aldosterone treatment induced a shift in band size for mMRE (-216T,-191C) compared to mMRE (-216G,-191C) or (-216G,-191A) (Fig. 1C). Incubation with nuclear extracts of MR-transfected HEK cells treated with aldosterone led to a distinct retention band for mMRE (-216T,-191C) but induced only a smear in mMRE (-216G,-191C) and (-216G,-191A), suggesting that MR interacts with mMRE (-216T,-191C) weakly in the absence of aldosterone but very strongly when aldosterone is present. Furthermore, the smear produced by other mMRE variants suggests either no binding of MR/SP1 or binding of a larger protein complex (Fig. 1D). Because SNP-191 (C/A) had no effect on MR-induced reporter gene activity or binding of SP1 under any of the different EMSA conditions we focused our further investigations on SNP-216 (G/T) (= rs712829).

To explore the effect of the two SNPs on MR-induced EGFR-promoter activity we performed reporter gene assays with mMRE reporters containing either the frequent allele mMRE (-216G) or the rare allele mMRE (-216T). MR activation led to a much higher stimulation of the mMRE (-216T) containing reporter than of the mMRE (-216G) variant in OK and HEK293 cells (Fig. 1E,F). The second SNP-191 (C/A) also located within the 65 bp of the minimal MR-responsive element but not in the SP1 binding region had no effect on the inducibility of the EGFR promoter fragment by MR activation (Fig. 1G,H).

### Influence of EGFR on cardiac gene expression

**Figure 2:**
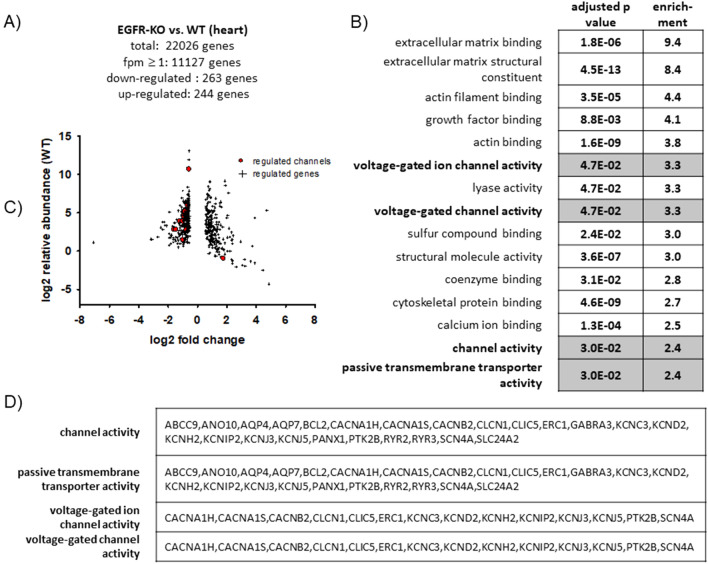
Influence of EGFR on the gene expression in heart tissue. (**A**) Next generation sequencing (NGS) experiments of heart tissue RNA of EGFR-KO and WT mice performed at the IZKF Leipzig Core Facility (Dr. Krohn) (N = 6). Of 22,026 genes, 11,127 were detectable with a mean fpm value of at least 1. 507 genes were regulated at least 1.5 fold and possessed a cohen´s d of ≥ 2 or ≤ 2 and were therefore considered relevantly regulated. Of those genes 263 genes were down-regulated and 244 were upregulated. (**B**) Functional enrichment analyses performed with the 507 relevantly regulated genes of our NGS analysis with G-profiler (https://biit.cs.ut.ee/gprofiler/gost): GO terms for molecular function with an adjusted *p* value of ≤ 0.05, at least twofold enrichment and an intersection size > 10 are listed with their respective adjusted p values and enrichment. Terms associated with transporter and channel activity are printed in bold. (**C**) Comparison of log2 relative abundance (WT) of wild type and log2 fold change (KO compared to WT) of all relevantly regulated genes of the NGS data with ion channels marked as circles. (**D**) List of regulated genes for all transporter- and channel- associated GO terms.

The EGFR is a known regulator of a multitude of pathways and genes and therefore an interesting signaling hub and therapeutic target. To explore the consequences of altered MR-induced EGFR expression for cardiac gene expression we performed next generation sequencing experiments of the transcriptome of heart tissue of EGFR WT and KO mice. Overall, 22,026 genes could be mapped, of which 263 were downregulated in the hearts of knockout (KO) compared to wild type (WT) mice and 244 genes were upregulated^28^. Most prominent enrichment was achieved for genes related to extracellular matrix components and cytoskeleton/actin binding which raises the possibility of involvement in structural remodeling processes of the heart, especially since *growth factor binding* was another enriched term. Cluster analysis also revealed overrepresentation of ion channels in the group of EGFR-regulated genes, which included subunits of T-type (CACNA1H) and L-type calcium channels (CACNA1S and CACNAB2) and several potassium channels as indicated in Fig. 2A-D (FPM ≥ 1, Cohen_d ≤ -2 ≥ 2, fold change ≥ 1.50 or ≤ 0.67).

**Figure 3:**
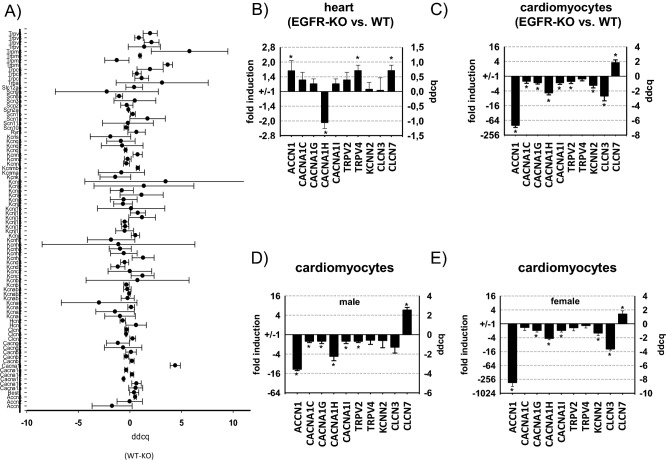
EGFR-dependent differential expression of ion channels in heart tissue and cardiomyocytes. (**A**) Detection of mRNA expression of 83 ion channels measured with qPCR arrays in heart tissue of EGFR-KO and WT mice and depicted as ΔΔCq values (ddcq) with confidence intervals (N = 2, n = 4; confidence interval of ΔΔct^EGFR-KO^ vs. ΔΔct^EGFR-WT^). (**B**) Validation of qPCR arrays by qPCR for the following ion channels: ACCN1 (amiloride-sensitive ion channel 1), CACNA1C, CACNA1G, CACNA1H, CACNA1I (voltage-dependent calcium channel subunit 1c/g/h/i/), TRPV2, TRPV4 (transient receptor potential cation channel, subfamily V, member 2/4), KCNN2 (small conductance calcium-activated potassium channel protein 2), CLCN3 AND CLCN7 (voltage-sensitive chloride channel 3/7) (N = 5–6, n = 10–12; data represented as mean ± SEM; *p ≤ 0.05). (**C**) Comparison of mRNA expression levels of different ion channels in freshly prepared cardiomyocytes of EGFR-KO and EGFR-WT mice (N = 10–11, n = 20–22; data represented as mean ± SEM; *p ≤ 0.05). (**D**–**E**) Comparison of ion channel expression measured by qPCR in freshly prepared cardiomyocytes of male EGFR-KO and WT mice (**D**) and female EGFR-KO and WT mice (**E**) (N = 4–6; n = 8–12; data represented as mean ± SEM; *p ≤ 0.05).

To look more specifically at regulation of ion channel expression by EGFR, RNA samples from hearts of EGFR-KO mice and WT control mice were further analyzed in an ion channel qPCR array that tested for 83 ion channel genes and used 5 housekeeping genes for standardization (Fig. 3A). Genes showing significant EGFR-dependent changes were selected and validated in hearts of EGFR KO and WT animals with qPCR using specific primers (Fig. 3B). CACNA1H was not included in our predesigned qPCR arrays but was added in our validation experiments because of the next generation sequencing results and the detection of two other T-type calcium channels, CACNA1G, AND CACNA1I, as EGFR-regulated genes in the qPCR arrays. Heart tissue of EGFR KO mice showed a significant increase in the expression of TRPV4, CLNC7 and a decrease in CACNA1H (Fig. 3B). Because heart tissue comprises of a mixture of different cell types including cardiomyocytes, fibroblasts as well as vascular and immune cells, we next isolated cardiomyocytes of EGFR KO mice and compared their ion channel gene expression to that of WT littermates. We found a reduced expression of ACCN1, CACNA1C, CACNA1G, CACNA1H and CACNA1I, TRPV2, KCNN2 and CLNC3 while CLCN7 was upregulated (Fig. 3C). For CLCN7 and CACNA1H expression changes in cardiomyocytes are more pronounced and go into the same direction as in heart tissue, indicating that the cardiomyocytes are the main site of expression. Only moderate sex-dependent effects were detected and for CACNA1H both male and female mice possessed a robust approximately fourfold reduction in gene expression in primary cardiomyocytes of EGFR KO mice compared to WT littermates (Fig. 3D-E).

### Involvement of EGFR in MR-induced expression of ion channels

**Figure 4:**
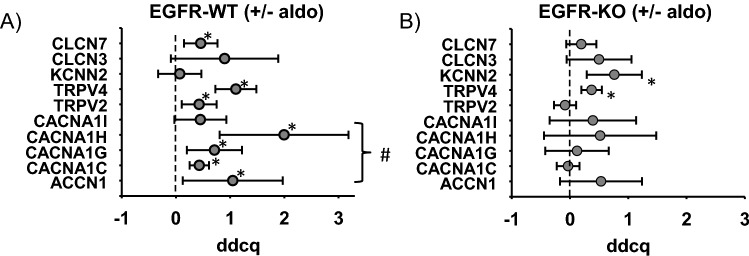
Aldosterone and EGFR-dependent differential expression of ion channels in cardiomyocytes. (**A**) Impact of in vivo aldosterone application on ion channel expression in freshly prepared cardiomyocytes of EGFR-WT mice determined by qPCR (ddcq = difference in quantitative cycles between normalized values of aldo and non-aldo animals) (N = 4–6; n = 8–12). All quantitative data represented as mean ± SEM; *p ≤ 0.05. EGFR-dependent aldosterone effects determined by comparison with results from EGFR-KO presented in (**B**) are labeled with (#). (**B**) Impact of in vivo aldosterone application on ion channel expression in freshly prepared cardiomyocytes of EGFR-KO mice determined by qPCR (ddcq = difference in quantitative cycles between normalized values of aldo and non-aldo animals) (N = 4–6; n = 8–12). All quantitative data represented as mean ± SEM; *p ≤ 0.05.

To test whether EGFR is also involved in MR-induced alterations of these ion channels in vivo, their expression was assessed in the heart tissue of EGFR-KO and WT type animals that received aldosterone or control treatment in vivo. Of the ion channels investigated, ACCN1, CACNA1C, CACNA1G, CACNA1H, TRPV2, TRPV4 AND CLCN7 were aldosterone-sensitive in their expression in the presence of EGFR in WT mice (Fig. 4A). CLCN3 expression possessed no aldosterone responsiveness. For KCNN2 and TRPV4 aldosterone regulation was EGFR-independent because it was also observed in EGFR KO mice (Fig. 4B). Therefore, we did not investigate it further. In contrast, the expression of CACNA1H, CACNA1G, CACNA1C, ACCN1 was only affected by aldosterone in WT animals. Because CACNA1H was a strongly EGFR-regulated ion channel in heart tissue and in cardiomyocytes and showed MR and EGFR-responsive regulation, our further investigations concentrated on this T-type calcium channel.

### Influence of EGFR activation on peak current density of I_Ca,T_ and CACNA1H protein expression in HL-1 cells

**Figure 5:**
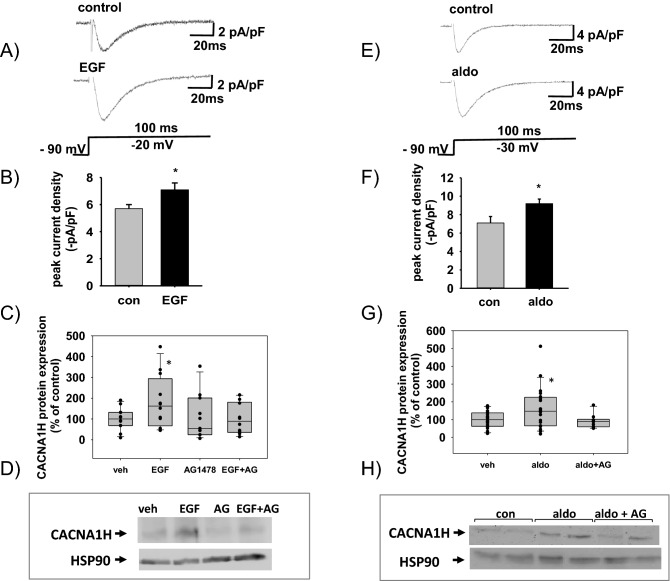
Effect of recombinant EGF, aldosterone and AG1478 treatment on peak current density of I_Ca,T_ and protein expression in HL-1 cells. (**A**) Comparison of characteristic original current traces induced by 100 ms long depolarizations from a holding potential of − 90 mV to a test potential of − 20 mV recorded under control condition (top panel) or after treatment of HL-1-cells with recombinant EGF for 48 h (lower panel). The corresponding pulse protocol is shown in the lower panel. Capacitive transients were partially removed for clarity. (**B**) Statistical evaluation of peak current density of I_Ca,T_ at a test potential of − 20 mV or − 30 mV recorded under control conditions (grey bar, N = 6, n = 59,) and after 48 h long incubation of HL-1 cells with recombinant EGF (black bar, N = 6, n = 46). (**C**) Quantification of protein expression of CACNA1H by Western blot after stimulation of HL-1 cells with vehicle, 10 µg/l EGF, 1 µM AG1478 or EGF and AG1478. *p ≤ 0.05. (**D**) Representative Western blot showing expression of CACNA1H after stimulation of HL-1 cells with vehicle, 10 µg/l EGF, 1 µM AG1478 or EGF and AG1478. (**E**) Comparison of typical original current traces induced by 100 ms long depolarizations from a holding potential of − 90 mV to a test potential of − 30 mV recorded under control condition (top panel) or after treatment of the cell with 10 nM aldosterone for 48 h (lower panel). (**F**) Statistical evaluation of peak current density of I_Ca,T_ at a test potential of -20 mV or -30 mV recorded under control conditions (grey bar, n = 31, N = 6) and after 48 h long incubation of HL-1 cells with 10 nM aldosterone (black bar, N = 6, n = 34). (**G**) Quantification of protein expression of CACNA1H by Western blot after stimulation of HL-1 cells with vehicle, 10 nM aldosterone or 1 µM AG1478 and aldosterone. *p ≤ 0.05. (**H**) Representative Western Blot showing expression of CACNA1H after stimulation of HL-1 cells with vehicle, 10 nM aldosterone or a combination of aldosterone and AG1478, *p ≤ 0.05.

We examined the effect of EGF on T-type calcium currents, I_Ca,T_ by treating cultured and serum starved HL-1 cells with recombinant 10 µg/l EGF for 48 h. From seven batches of cells tested, 6 responded to application of EGF with an increase of I_Ca,T_. Figure 5A shows a representative I_Ca,T_ recorded under control conditions with a peak current density of -4.6 pA/pF in comparison to a representative recording after EGF treatment with a peak current density of -7.0 pA/pF. On average control peak current density of I_Ca,T_ amounted to -5.7 pA/pF ± 0.3 pA/pF (n = 59), while after EGF treatment a mean peak current density of -7.1 pA/pF ± 0.5 pA/pF (n = 46) was recorded (Fig. 5B). The T-type calcium channel antagonist ML218 was able to abolish I_CaT_ currents and this effect could be reversed through a wash out phase (Supplementary Fig. S1). No change in activation or inactivation of T-type calcium current was found (Supplementary Fig. S1). Furthermore, incubation of HL-1 cells with EGF increased protein expression of CACNA1H as shown by a representative Western blot and protein quantification (Fig. 5C,D). This increase could be attenuated by the EGFR tyrosine kinase inhibitor AG1478 which on its own did not exert any effect on CACNA1H expression. Figure 5E,F show the effect of 10 nM aldosterone on peak current density of I_CaT_. Aldosterone incubation increased mean peak current density significantly from -7.1 pA/pF ± 0.7 pA/pF (n = 31) to -9.2 pA/pF ± 0.5 pA/pF (n = 34). Western blot analysis of lysates of HL-1 cells incubated with 10 nM aldosterone also showed an increase in CACNA1H protein expression, which could be inhibited by AG1478 (1 µM), suggesting that EGFR mediates the aldosterone/MR effect on CACNA1H (Fig. 5G,H). Preliminary data from rat neonatal cardiomyocytes also show that 10 nM aldosterone and 10 µg/l EGF enhance CACNA1H protein expression, while 1 µM AG1478 can inhibit the effect of aldosterone (Supplementary Fig. S3).

### Relevance of enhanced CACNA1H activity in HL-1 cells

**Figure 6:**
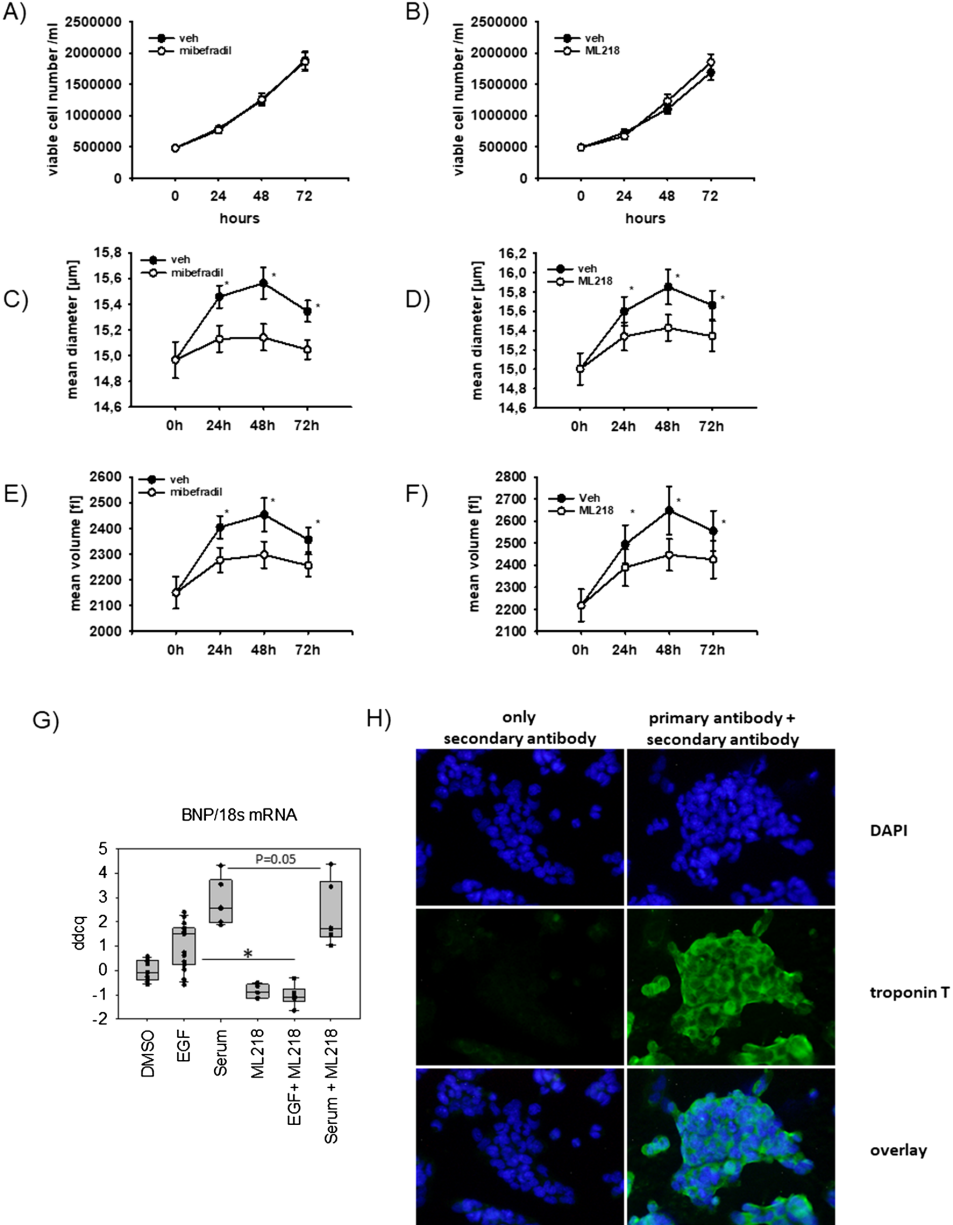
Effect of T-type calcium channel inhibitors on cell proliferation and mean cellular diameter and cell volume of HL-1 cells. (**A** + **B**) Assessment of cell proliferation of HL-1 cells over 72 h in the presence and absence of T-type calcium channel inhibitors. 300,000 HL-1 cells were seeded in 3 cm petri dishes and cultivated in Claycomb medium with serum for 24 h prior to measurements. Proliferation of cells was measured after 0, 24, 48 or 72 h by counting viable cells with a Casy Cell Counter and Analyzer. Cells were either incubated with vehicle (veh), 1 µM ML218 (**A**) or 1 µM mibefradil (**B**), two T-type calcium channel inhibitor (N = 6; n = 11–12 for mibefradil and control, N = 9; n = 17–18 for ML218 and control). All quantitative data represented as mean ± SEM; *p ≤ 0.05. (**C**-**F**) Determination of mean cell diameter and volume in HL-1 cells by resistance measurements with a Casy Cell Counter and Analyzer. Mean cell diameter (**C** + **D**) and mean volume (**E** + **F**) were quantified in HL-1 cells as indicated. (N = 6; n = 11–12 for mibefradil and control, N = 9; n = 17–18 for ML218 and control). All quantitative data represented as mean ± SEM; * p ≤ 0.05. (**G**) As marker for cardiomyocyte hypertrophy, BNP mRNA levels were quantified in HL-1 cells incubated with either vehicle (DMSO), 10 µg/l EGF, 10% serum, 1 µM ML218 or combinations thereof, n = 6–16, *p ≤ 0.05. (**H**) Representative immunofluorescence images of HL-1 cells stained with DAPI and troponin T primary antibody and secondary antibody or only secondary antibody.

To assess the biological relevance of changes in CACNA1H activity, the effect of two T-type calcium inhibitors mibefradil and ML218 on cell proliferation and cell volume was analyzed in HL-1 cells cultivated in serum-containing media. Of the two inhibitors, ML218 is more specific and does not affect L- and N-type calcium channel activity. Over the time course of 3 days, cell numbers increased to the same degree in cells treated with vehicle or either of the two T-type calcium channel inhibitors (3.90 ± 0.47 and 3.86 ± 0.47 fold change for mibefradil and 3.44 ± 0.43 and 3.77 ± 0.46 fold change for ML218 respectively) (Fig. 6A,B). When looking at cell diameter and volume of these HL-1 cells, we observed that incubation with calcium channel inhibitors led to a decrease in cell diameter and volume (Fig. 6C,F). HL-1 cells had an initial mean diameter of 14.97 ± 0.14 and 15.00 ± 0.16 µm and showed an increase within the first 48 h of incubation time to 15.85 ± 0.12 and 15.56 ± 0.18 µm respectively. This increase in cell diameter was diminished by mibefradil by 70% and by ML218 it was reduced by 50%. This suggests that calcium channels support cellular hypertrophy (Fig. 6C,F). Likewise, qPCR analysis revealed increased BNP levels with EGF and serum that could be inhibited by ML218 (Fig. 6G). Figure 6H depicts an immunofluorescence stainings of HL-1 cells with the cardiomyocytes marker troponin T.

**Figure 7:**
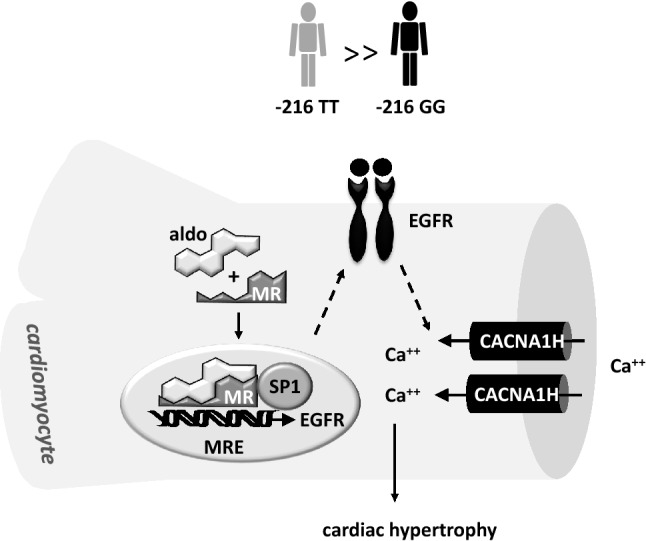
Proposed model linking cardiac MR activation and SNP-216 (T/T) to enhanced EGFR and CACNA1H expression and changes in cardiomyocyte volume.

In summary, our results indicate that aldo/MR via the EGFR regulates the expression of ion channels in cardiomyocytes, especially of CACNA1H. An increase in CACNA1H expression is associated with an increase in cell diameter and volume, indicating it may facilitate cellular hypertrophy as suggested by our BNP measurements (Fig. 7). Consequently, through this pathway the MR may mediate part of its pathological effects in the cardiovascular system. A SNP within the MR responsive element has a large impact on the magnitude of the MR-induced EGFR expression. It is tempting to speculate that by genotyping, a cohort of patients especially susceptible to pathological MR effects and therefore responsive to MR antagonists can be identified.

## Discussion

Large clinical studies like RALES, EPHESUS and EMPHASIS-HF demonstrate a reduction in mortality and hospitalization rate of cardiovascular patients after treatment with MR antagonists independently of blood pressure or electrolyte homeostasis^1,2,29–31^. Community-based samples and patient data indicate a correlation between aldosterone-renin ratios and structural left ventricular (LV) remodeling, which is a strong risk factor for poor outcome in cardiac diseases^32,33^. In clinical trials patients with LV remodeling that received MR antagonists showed a reduction in LV mass and improved LV function that cannot be fully explained by a reduction in blood pressure^34^. In animal studies, the MR antagonist eplerenone also improved LV remodeling after extensive myocardial infarction with less fibrosis and cardiac hypertrophy^35^. Cardiac remodeling was attenuated by cardiomyocyte-specific MR deletion but not by deletion of MR in cardiac fibroblasts, demonstrating the importance of cardiomyocytes for this process^36,37^. The exact molecular mechanisms leading to pathological cardiac MR effects are not clear.

In previous work, we and others found that the MR can increase EGFR expression and signaling^27,38,39^. In the cardiovascular system, the EGFR functions as a central hub for different signaling cascades and therefore its expression can affect a wide variety of signaling pathways. In our investigation, the EGFR promotor activity induced by aldosterone/MR was strongly influenced by the promoter polymorphism -216 (G/T). For both aldosterone/MR and the EGFR, a stimulatory effect on cardiac hypertrophy and remodeling has been demonstrated. As underlying mechanism, aldosterone/MR can rapidly transactivate the EGFR but they can also stimulate EGFR promotor activity by acting as a transcription factor^27,40^. Several studies report that in different cancer cell lines the -216T/T genotype of the EGFR promoter leads to the highest EGFR expression and the -216G/G genotype to the lowest with the -216 G/T genotype being in between. Additionally, they find a higher affinity of nuclear proteins and SP1 to the -216T probe compared to the -216G probe in EMSAs^41–43^. In literature, this polymorphism has already been linked to altered EGFR expression in human fibroblasts and bronchial epithelial cells. Additionally, the -216T/T and G/T phenotype was more frequent in lung cancer patients with pleural metastasis compared to a non-metastasis group. Patients with primary lung adenocarcinoma with -216T/T and -216G/T had a higher EGFR expression compared to those with-216G/G^41–43^.

Participation of the EGFR in cardiovascular pathology has been reported in literature and supports a possible role in pathological MR effects. It has been shown that in vivo application of EGFR inhibitors or antisense oligonucleotides can prevent hypertension and cardiac hypertrophy in different animal models^44–47^. For erlotinib, the EGFR inhibitor AG1478 or wav-2 mice, a reduction in vascular hypertrophy and perivascular fibrosis was reported in angiotensin II-infused mice^48,49^. In cardiomyocytes, EGFR transactivation by angiotensin II led to cellular hypertrophy, which could be prevented by the EGFR inhibitor AG1478 or by dominant negative Gαq^50^. The MR inhibitor canrenoate was also able to reduce cardiac hypertrophy that was induced by angiotensin II^51^. Although Messaoudi found that aldosterone-induced cardiac hypertrophy was not prevented in a heart-specific dominant negative EGFR transgenic mouse^50^ others found that aldosterone can stimulate NHE1 via EGFR transactivation and thereby induce cardiac hypertrophy^52^. Activated NHE1 can increase the sodium content of cells, causing augmented Na^+^-Ca^++^-transporter activity and calcium content of the cells, which enhances contractility and hypertrophy. Aldosterone has been also shown to EGFR-dependently  activate ERK1/2 and p90RSK, which have been implicated in cardiac hypertrophy^52^. EGFR transactivation by aldosterone stimulated Nox-dependent ROS generation, which also can support cardiac hypertrophy^53^. For the EGFR it has also been shown that it can be transactivated by G protein-coupled receptors of vasoactive peptides like angiotensin II or catecholamines through activation of matrix metalloproteases like ADAM 12, which then lead to shedding of membrane-bound HB-EGF^54^. In cardiomyocytes, enhanced EGFR expression can lead to cardiomyocyte hypertrophy via a G protein dependent pathway but also to cardiomyocyte survival via a G protein-independent beta arrestin-dependent pathway^55^. An increase in EGFR expression through aldosterone/MR would mean that other hypertrophic stimuli would be able to cause more EGFR transactivation and downstream hypertrophic signaling. There are reports that the EGFR can additionally enter the nucleus as a transcription factor. Further experiments are required in the future to determine the prevalent mechanisms in different pathophysiological contexts.

In our experiments, we searched for gene clusters regulated by EGFR that could mediate pathological MR effects in the heart. In our cluster analysis of EGFR KO mice compared to wild type littermates we found that ion channels and especially voltage gated channels were regulated by EGFR. In our NGS transcriptome and qPCR array analysis, T-type calcium channels and especially CACNA1H was shown to be EGFR-dependently regulated in the heart and in cardiomyocytes. Other channels like the L-type calcium channel pore forming unit CACNA1C were also differentially regulated in EGFR-KO and WT animals and were influenced in their expression by aldosterone. However, the channel which was the most strongly and stably regulated in all experimental designs was CACNA1H (Cav3.2) and therefore, we focused on it in further analyses. Additionally, two other T-type calcium channels were regulated in an EGFR- and aldosterone-dependent manner, suggesting T-type calcium channels in general might be of importance. CACNA1C was regulated to a much lesser extent and less robustly especially when comparing data from NGS and qPCR arrays. Nevertheless, enhanced T-type calcium channel expression may lead to depolarization and activation of L-type calcium channels to further induce cardiac hypertrophy especially when L-type calcium channel subunit expression is increased. A role for CACNA1C for cardiac hypertrophy has been previously reported^56^. Other ion channel-mediated currents for which an effect of EGFR has been reported in literature include HCN channel currents^57,58^, potassium currents mediated through phosphorylation of Kv4.3, HERG (Kv11.1) and KCNQ1/KCNE1^59–61^ and reperfusion arrhythmias in anaesthetized rats caused by phosphorylation of L-type calcium and sodium channels^62,63^.

Global EGFR-KO mice either die at midgestation due to placenta defects or the mice live up to 3 weeks but display various abnormalities depending on genetic background. However, they have no gross differences in heart histology compared to WT littermates^64^. The phenotype of mice with reduced EGFR tyrosine kinase activity (wa-2 mice) is also variable depending on mouse strain. They suffer mainly from abnormal valve formation and aortic stenosis but only C57B6J mice with reduced EGFR activity develop cardiac hypertrophy^65^. A knock-in rescue of EGFR-KO mice with higher than normal expression of EGFR resulted in a twofold increased heart weight with severe hypertrophy of the left ventricular wall and interventricular septum and cardiomyocyte hypertrophy. SM22-EGFR-KO mice have a deletion of the EGFR in arterial VSMCs and in cardiomyocytes. They possess a reduced life span, normal systolic blood pressure but reduced diastolic blood pressure and total peripherial resistance compared to WT animals. They also have a ventricular hypertrophy with increased stroke volume that is already present in newborn mice^66^. Overall, deciphering the role of cardiac EGFR is complicated with the existing models because of its dual importance first for cardiovascular development and later in life for pathophysiological changes.

By in vivo application of aldosterone or vehicle to EGFR KO and WT animals and subsequent analysis of freshly prepared cardiomyocytes, the importance of EGFR per se and for aldosterone-induced upregulation of CACNA1H could be demonstrated. For many ion channels there were differences in the regulation of their expression in murine heart tissue and in isolated cardiomyocytes. One explanation for this is that cardiac tissue only contains approximately 30% cardiomyocytes. The rest of the cells consist mainly of cardiac fibroblasts followed by endothelial cells, VSMC and immune cells so that expression changes in cardiomyocytes might be blunted by the other cells^67–69^. Consequently, in non-cardiomyocyte cells of the heart, availability of ligands and signaling molecules and therefore regulation of EGFR and ion channels may vary. Additionally, heart tissue is influenced by extracellular fluid and blood concentrations as well as adjacent cells while isolated cardiomyocytes miss these influences and may also change their gene expression profile during the isolation and cultivation process.

We were able to confirm our results for MR- and EGFR-regulated upregulation of CACNA1H from animal studies in HL-1 cells by showing that stimulation with EGF or aldosterone enhances CACNA1H protein expression and T-type calcium peak current density. HL-1 cells are derived from the AT-1 mouse atrial cardiomyocyte tumor lineage. They reportedly possess a gene expression similar to that of adult atrial myocytes even after passaging, including adult isoforms of α-cardiac myosin heavy chain and cardiac actin as well as connexin43^70^. Furthermore, HL-1 cells possess the advantage that they express CACNA1H under basal conditions, which healthy adult ventricular cardiomyocytes do almost not. To minmize changes in phenotype we passaged HL-1 cells a maximum of 10 times. In HL-1 cells, aldosterone induced increase in protein expression of CACNA1H could be attenuated by the EGFR inhibitor AG1478. These findings are in good agreement with the results of other groups that reported an increase in T-type calcium channels mediated by aldosterone in HL-1 cells, rat cardiomyocytes and in adrenocarcinoma cells (H295R)^71–73^. Data from KO mice suggest that CACNA1H expression does not influence aldosterone levels and also has no effect on angII-dependent hypertension and therefore seems to affect remodeling processes directly^51^.

T-type calcium channels are low-voltage-activated channels that are expressed throughout the nervous, the neuroendocrine and the cardiovascular system. Voltage-dependent opening of T-type channels occurs at comparatively negative membrane potentials where calcium influx contributes to the depolarization of the plasma membrane and increases the opening probability of other voltage-gated channels. Physiologically, in the cardiovascular system T‐type Ca^2^^+^ channels are involved in the maintenance of vascular tone and cardiac automaticity and they do not seem to contribute significantly to myocardial contraction. There are two cardiac isoforms CACNA1G and CACNA1H. Both forms are mainly expressed in pacemaker tissues but also in atrial and ventricular myocytes especially under pathological conditions^74^. In the heart, CACNA1G is the predominant form in adults while CACNA1H is highly expressed in the embryonic period. However, recent studies indicate that T-type calcium channels reappear in myocardium with LV hypertrophy or cardiac failure and under certain neurohumoral stimulations^75–78^. The re-expression of ventricular T-type channels observed in wild-type hypertrophic hearts was absent in CACNA1H-null mice, which suggests that re-expressed T-type calcium channel is encoded by CACNA1H. Based on data from CACNA1H-KO mice that have a normal heart rate without arrhythmias, CACNA1H encoded channels do not seem to be involved in generation of pacemaker potentials^79^. Instead, re-expressed CACNA1H protein might induce cardiac hypertrophy by activating the calcineurin–NFAT pathway^80^. CACNA1H expressed T-type calcium channels have been shown to associate with calcineurin^81^ and thus could play a role in the hypertrophic signaling. Furthermore, CACNA1H was required for angiotensin II- and pressure overload-induced cardiac hypertrophy in mice through activation of calcineurin/NFAT^80,82,83^. AngII-increase in blood pressure was not CACNA1H-dependent^51^. On the other hand, cardiac hypertrophy seems to induce CACNA1H expression by Egr1 and CACNA1H is an important mediator of Egr1 in regulating cardiac hypertrophy leading to a circulus vitiosus^84^. In our experiments, inhibition of T-type calcium channels by either mibefradil, which is a 5–10 times more potent blocker of T-type than of L-type calcium channels, or the more specific ML218 was able to reduce the cell size of HL-1 cells suggesting that T-type calcium channels may play a role in cardiomyocyte hypertrophy and cardiac remodeling by affecting intracellular calcium homeostasis. In literature, compared to classical L‐type Ca^2^^+^ channel antagonists, mibefradil appears beneficial in animal models of hypertension and heart failure to prevent cardiorenal injury and to improve survival rates^85^. Mibefradil partly restored the positive inotropic response to beta-adrenergic stimulation in hypertrophied myocardium from aortic-banded rats, an effect that might be useful in hypertrophied myocardium with impaired intracellular Ca^2^^+^ homeostasis^86^. Furthermore, CACNA1H channels have been associated with atrial remodeling and increased vulnerability and duration of atrial fibrillation^87,88^. Moreover, a pharmacological blockade of T-type calcium channels reduced arrhythmias during dilated cardiomyopathy and prevented sudden death due to myocardial infarction in mice. While CACNA1H seems to be involved in the induction of heart hypertrophy, some data suggest CACNA1G may be protective of heart hypertrophy^89^. While mice lacking CACNA1H showed reduced cardiac hypertrophy after pressure overload^90^, mice overexpressing CACNA1G were somewhat protected from hypertrophy despite increased calcium transients and sarcoplasmatic calcium load with enhanced contractile function^89^. CACNA1G KO animals mainly display a reduction in heart rate and pacemaker activity with an increased susceptibility to hypertrophic response. Despite having similar current characteristics, CACNA1G and CACNA1H have differences in structural and kinetic features and functions^91,92^ and are regulated differently for example by calcium feedback and CAMKII^93,94^. One explanation for these different findings are alternative signaling domains of the two receptor subtypes or altered signal coupling in the same microdomain^95^. Antihypertrophic effects of CACNA1G signaling may be mediated by eNOS. In CACNA1H KO mice, TAC led to a less pronounced hypertrophic response and re-expression of CACNA1H had a prohypertrophic effect via the NFAT calcineurin signaling pathway. Consequently, CACNA1G seems to influence heart rate while CACNA1H seems to be involved in cardiac hypertrophy^96,97^. In the vasculature, CACNA1G and CACNA1H also have varying function. CACNA1H activation leads to dilation of blood vessels, whereas CACNA1G mainly affects constriction but is also involved in neointima formation following vascular damage.

In our investigation we used heart tissue and isolated cardiomyocytes of SM22-EGFR KO mice and controls treated in vivo with aldosterone for 28 days, HL-1 cells and rat neonatal cardiomyocytes incubated for 48–72 h as models. Finding an adequate model to study CACNA1H is challenging. Global EGFR-KO mice exibit a phenotype that is highly dependent on the genetic background. To test the effect of EGFR in adult hearts and to avoid a lethal phenotype we utilized a conditional EGFR-KO in cardiomyocytes and VSMCs. However, it is not clear if the cardiac phenotype is the result of a developmental problem or the postnatal loss of EGFR in cardiomyocytes or VSMCs or a response to the changes in total peripheral resistance and blood pressure. To limit the number of animals sacrificed and for mechanistic studies primary cells or cell lines are commonly used. In our case, the choice of a model to study MR, EGFR and CACNA1H in cardiomyocytes is challenging. Using murine cardiomyocytes of SM22-EGFR KO mice and controls for further investigations is problematic because of possible developmental defect they may possess and because in healthy adult WT mice CACNA1H is only expressed in the electrical conductance system and the atria and hardly at all in the contractile ventricular cardiomyocytes. A re-expression only takes place after pathological stimulation^92^. In general, cultivating primary murine cardiomyocytes and incubating them for 48 h ex vivo leads to loss of their typical properties and cell death. As a compromise we employed HL-1 cells, which express CACNA1H and can be cultivated for longer but are an atrial cell line. In addition, neonatal rat cardiomyocytes were used to support our findings in preliminary experiments. Cardiomyocytes from inducible cardiomyocyte-specific EGFR KO mice that were treated in vivo with stimulants like aldosterone would be a better choice or human heart tissue samples of people with elevated aldosterone plasma levels and with known clinical data like echocardiography. However, establishing additional new genetic models and performing in vivo experiments or collecting patient samples is beyond the scope of this report but will be considered for future investigations.

Overall, our results indicate that the previously described regulation of EGFR expression by MR and SP1 is modulated by SNP -216(G/T) on the EGFR promoter, which may help identify patients that will benefit most from MR antagonists and are the most prone to cardiovascular remodeling induced by vasoactive substances that can transactivate the EGFR. The interaction between MR and EGFR led to enhanced expression of different ion channels of which the T-type calcium channel CACNA1H was the most stable and prominent. An EGFR-dependent MR-mediated increase in protein expression of CACNA1H with augmented T-type calcium peak currents was confirmed in HL-1 cells incubated with aldosterone, EGF and the EGFR inhibitor AG1478. HL-1 cells also showed reduced cardiomyocyte diameters and BNP levels after incubation with T-type calcium channel inhibitors, suggesting that MR via EGFR and CACNA1H expression modulates cardiomyocyte growth and thereby promotes cardiac remodeling processes.

## Methods

### Cell culture

HEK-293 cells were acquired from ATCC (Rockville, MD) and cultivated in DMEM/Ham´s F-12 medium supplemented with 10% fetal calf serum. HEK cells do not possess functional endogenous MR. OK proximal tubule cells from opossum (Dr. Biber, University of Zurich) were grown in MEM medium, pH 7.4, supplemented with Earl’s salts, non-essential amino acids and 10% fetal calf serum. Transient transfections were performed with Polyfect Reagent (Qiagen; Hilden, Germany) for HEK cells and Effectene (Qiagen; Hilden, Germany) for OK cells, according to the manufacturer´s instructions in medium without supplements. HL-1 cardiomyocytes were a kind gift of Prof. Dr. William C. Claycomb (LSU Health Sciences Center; New Orleans, USA) and cultivated in Claycomb medium (Sigma-Aldrich; Munich, Germany) supplemented with 10% fetal bovine serum, penicillin (100 U/ml)/streptavidin (100 μg/ml), norepinephrine (0.1 mM) and L-glutamine (2 mM). Cells were cultured on pre-coated flasks and dishes with 12.5 µg/ml fibronectin (BD Biosciences, PA) and 0.02% gelatin (Sigma-Aldrich) in a humidified atmosphere of 5% CO_2_ at 37 °C. Prior to experiments, cells were made quiescent by incubating them for at least 24 h in medium without serum and steroids.

Neonatal rat cardiomyocytes were bought from Lonza (R-CM-561). Cells were cultivated according to the manufacturer’s instructions in RCGM Rat Cardiomyocyte Growth Medium with gentamicin/amphotericin and 10% horse serum. For experiments, medium without growth factors and serum was used.

### Plasmids

pEGFP-MR is a kind gift of N. Farman (Paris). To exclude that the EGFP-tag changes the properties of the human mineralocorticoid receptor (hMR), we compared its GRE activation and nuclear transcription with that of untagged hMR. No significant changes were detected^40^. pcDNAHisLacZ was purchased from Invitrogen (Life Technologies; Darmstadt, Germany). The secretory alkaline phosphatase (SEAP) was used as a reporter gene, which is encoded on a pSEAP2-basic vector from Clontech (Mountain View, CA, USA). EGFR promoter constructs were cloned into pSEAP2-basic by PCR cloning using T4 DNA Ligase (Invitrogen/Life technologies; Darmstadt, Germany). To confirm sequence identity all promoter fragments were sequenced (Eurofins MWG Operon, Ebersberg, Germany).

### Reporter gene assays

Reporter gene assays were performed with HEK-293 or OK cells, which were transiently co-transfected with hMR, pcDNA-HisLacZ (internal control) and EGFR-promoter-SEAP constructs or empty vector. SEAP activity was detected by fluorescence measurements with the AttoPhos System (Promega; Mannheim, Germany). Beta-galactosidase was measured at 405 nm by colorimetric assay with ortho-nitrophenyl-galactosidase as substrate.

### EMSA

Electromobility shift assays were performed with the Gelshift Chemiluminescent EMSA Kit (Active Motif; Rixensart, Belgium) and biotinylated probes assembled by oligo hybridization or PCR using biotinylated dUTPs (for oligonucleotides see Supplentary Fig. S4). Binding reactions were carried out for 30 min at room temperature in the presence of 10 mM Tris, 50 mM KCl, 5 mM MgCl_2_, 1 mM DTT, 5% glycerol and 1 mg/ml poly(dI-dC). 5 fmol of biotinylated probe per reaction were incubated with 300 ng rhSP1 (Promega; Mannheim, Germany), 150 ng rhSP3 (Abnova; Taiwan) or appropriate amounts of BSA as negative control. Nuclear extracts were isolated from HEK293 cells with the Nuclear Extraction Kit (Active Motif; Rixensart, Belgium) and desalted with centrifugal filter units (Millipore; Billerica, USA). Electrophoresis was carried out using a 10% non-denaturing polyacrylamide gel at 140 V for 2 h. Samples then were transferred to a nylon transfer membrane (Whatman/GE Healthcare Life Sciences; Freiburg, Germany) at 250 mA for 45 min and cross-linked for 1 min at 120 mJ/cm^2^. A detailed view of the EMSAs is provided in Supplementary Fig. S7.

### EGFR KO mice

C57BL/6 mice with floxed EGFR alleles were obtained from Dr. M. Sibilia (University of Vienna) and used for further breeding. EGFR was tissue specifically inactivated in cardiomyocytes and smooth muscle cells using transgenic SM22-Cre mice. All mouse experiments described in this manuscript were approved by the local government (Landesverwaltungsamt Sachsen-Anhalt, Germany) and were performed according to the guidelines of the directive 2010/63/EU. Mice were kept in the facilities of the University of Halle-Wittenberg at a room temperature of 20 ± 1 °C and with a 12 h/12 h light/dark cycle. In vivo application of aldosterone with salt for 28 days with the help of a subcutaneous pellet was described previously in accordance with the ARRIVE guidelines^98^.

### Cardiomyocyte isolation

Cardiac myocytes were isolated by collagenase dissociation technique as described previously^66^. Using a Langendorff apparatus, excised hearts were retrogradly perfused with Tyrode solution (mmol/L: 150 NaCl, 5.4 KCl, 20 glucose, 20 2,3-butanedione monoxime, 5 HEPES; pH 7.4;37 °C; 2 ml/ min): Tyrode solution supplemented with CaCl_2_ (1.8 mmol/L) for 4 min, nominally Ca^2^^+^-free Tyrode solution for 10 min, nominally Ca^2^^+^-free Tyrode solution supplemented with collagenase Type II (60U/mL, Worthington) and protease Type XIV (0.3 U/mL) for 10 min, Tyrode solution supplemented with CaCl_2_ (90 μmol/L) for 10 min were applied. Subsequently, ventricles were cut and myocytes were dissociated into Tyrode solution supplemented with CaCl_2_ (90 μmol/L). Cells were gently centrifuged (20 g, 3 min) and the supernatent was discarded. Cardiomyocytes were allowed to settle by gravity to a laminin covered plate (2 h); afterwards the supernatent was removed.

### Analysis of mRNA expression by next generation sequencing

Total RNA from hearts of six EGFR KO and six WT littermates was isolated using TRIzol Reagent. Library preparation and next generation sequencing (Illumina HiScan Run 2 × 100 bp) was commercially performed by the IZKF Leipzig (Prof. Krohn). Data were analyzed using g:Profiler (http://biit.cs.ut.ee/gprofiler/) and GOrilla (cbl-gorilla.cs.technion.ac.il).

### RT^2^ profiler PCR arrays

Total RNA from EGFR KO and EGFR wildtype mice was isolated with TRIzol Reagent, converted to cDNA and used for RT^2^ Profiler PCR Arrays (Qiagen, Hilden, Germany).

### Quantification of mRNA by qPCR

Total RNA from EGFR KO and EGFR wildtype mice treated with or without aldosterone and salt was isolated using InviTrap Spin Tissue RNA Mini Kit (STRATEC Molecular; Berlin, Germany). HL-1 cell RNA was isolated with TRIzol Reagent according to the manufacturer´s instructions. RNA was subject to DNAse digestion and reverse transcription with SuperScript II Reverse Transcriptase (Thermo Fisher Scientific, Waltham, MA, USA) and reverse primers. Real time PCR was performed with Platinum SYBR Green qPCR Supermix in a 7900HT Fast Real‐time PCR system (Thermo Fisher Scientific, Waltham, MA, USA). Equal amounts of cDNA were analyzed by qPCR using the delta-deltaCT method. Primers are listed in Supplemental Figs. S5 and S6.

### Electrophysiological recordings

HL-1 cells were cultivated in Claycomb medium. One day after subculture the cells were serum starved for 24 h before EGF (10 µg/ml) was added. Electrophysiological recordings were made 48 h post drug application in the whole-cell configuration of the patch-clamp technique with an Axopatch 200A patch-clamp amplifier (Axon Instruments, Inc., Burlingame, CA, USA).

Patch pipettes were pulled from thick wall borosilicate glass (Hilgenberg, Malsfeld, Germany). Electrical resistances of fire polished electrodes were 3-4MΩ when filled with the pipette solution containing (in mmol/L) 130 CsCl, 20 TEACl, 10 EGTA, 5 Na_2_ATP, 6 MgCl_2_, 10 HEPES, pH 7.2 (adjusted with CsOH). The Na^+^-free and K^+^-free bath solution for whole-cell recordings consisted of (in mmol/L) 150 Tris-Cl, 1.8 CaCl_2_, 10 glucose, 10 HEPES, pH 7.4 (adjusted with Tris-OH). Membrane currents were low pass filtered at 5 kHz with an eight-pole Bessel filter built in the amplifier, digitized at 16 or 40 kHz and stored for off-line analysis (ISO2, MFK, Germany). Series resistance was routinely compensated by > 70%. The input capacitance of HL-1 cells was determined by integrating the capacitive current at the end of a 10 ms long voltage step from − 60 to − 50 mV. Peak current amplitudes were normalized to this value to obtain current densities (pA/pF) in order to reduce variability due to cell size. All measurements were performed at room temperature (20–24 °C). No correction for liquid junction potentials was applied. Since the aim of this study was to investigate the effect of EGF exclusively on the activity of T-type calcium channels, we used cells that either had alone I_Ca,T_ or cells in which the contribution of I_Ca,L_ to the inward current was not detectable at the test potential. To maximally activate I_Ca,T_ single HL-1 cells were depolarized every 8 s for 100 ms from a holding potential of − 90 mV to test potentials of − 20 mV and − 30 mV to obtain the maximal peak inward current. Current amplitudes were determined as difference between peak I_CaT_ and currents recorded at the end of the depolarizing voltage step. Time to half maximal activation (t_1/2_ activation) and time to 33% inactivation (t_1/3_ inactivation) were measured after depolarizing cells from a holding potential of − 90 mV to a test potential of − 30 mV (Supplementary Fig. S2).

### Western blot

HL-1 cells were lysed in RIPA buffer (150 mM NaCl, 10 mM Tris pH 7.4, 1% Nonidet P-40, 0.1% SDS, 1% sodium deoxycholate, 0.1% Triton-X, 1 mM EDTA, sodium orthovanadate, 1 mM NaF, protease inhibitor cocktail), separated by 8% SDS-PAGE and transferred to nitrocellulose membrane. Membranes were incubated with anti-CACNA1H (Novus Biologicals; Littleton, CO, USA) and anti-HSP90 (Cell Signaling Technology/NEB, Frankfurt, Germany). The bound primary antibody was visualized using horseradish peroxidase-conjugated secondary IgG and the Immun-Star WesternC Chemiluminescent Kit. The signal was detected with the chemiluminescence detection system in the linear detection range. Densitometry analysis was performed with Quantity One (Bio-Rad; Munich, Germany). A detailed view of the Western Blots of Fig. 5 is provided in Supplementary Fig. S8.

### Cell count and cell diameter

HL-1 cells were seeded in petri dishes and analyzed 0, 24, 48 and 72 h after incubation with either vehicle, 1 µM ML218 or 1 µM mibefradil in Claycomb medium. At the appropriate time points, cells were trypsinized and cell suspensions were analyzed with Casy cell sorter and analyzer.

### Immunofluorescence microscopy

HL-1 cells were cultivated on poly-l-lysine-coated 10-well-glass cover slips and were fixed with 4% paraformaldehyde (15 min), washed three times with PBS and permeabilized with 0.5% Triton-X100 (30 min). Subsequently, cells were incubated for 10 min in 1% SDS/PBS/100 mM glycine, followed by 10 min 100 mM glycine/PBS and finally for 20 min in 10% serum/1% BSA/PBS. After overnight incubation with anti-troponinT antibody (Cell Signaling 1:300), the cells were washed three times with PBS and incubated for 45 min with oregon green coupled secondary antibody. After three more washes with PBS, cell nucli were stained with DAPI (1 μg/ml). Cells were analyzed using the Keyence Fluorescence Microscope BZ‐8100E (Keyence, Osaka, Japan).

### Statistics

Significance of difference was tested by Student´s t-test, one-way anova or confidence interval with *p* ≤ 0.05 considered statistically significant. N represents the number of subjects, animals or experiments and n the number of wells or culture dishes. Analysis of the data of the electrophysiological recordings was performed using Origin 8.0 (Microcal, Northampton, MA, USA).

## Supplementary Information

Supplementary Information.
