# Supplementary material for: The mineralocorticoid receptor leads to increased expression of EGFR and T-type calcium channels that support HL-1 cell hypertrophy
Source: Sci Rep. 2021 Jun 24;11:13229. doi: 10.1038/s41598-021-92284-y (PMC8225817; doi:10.1038/s41598-021-92284-y)
Supplement: Supplementary file 1 — Supplementary Information. [file 41598_2021_92284_MOESM1_ESM.pdf]

The Mineralocorticoid Receptor Leads to Increased Expression of EGFR and T-Type Calcium Channels that Support HL-1 Cell Hypertrophy

Katharina Stroedecke[1+], Sandra Meinel[1#+], Fritz Markwardt[1], Udo Kloeckner[1], Nicole Straetz[1], Katja Quarch[1], Barbara Schreier[1], Michael Kopf[1], Michael Gekle[1], Claudia Grossmann[1*]

Suppl. Fig. 1

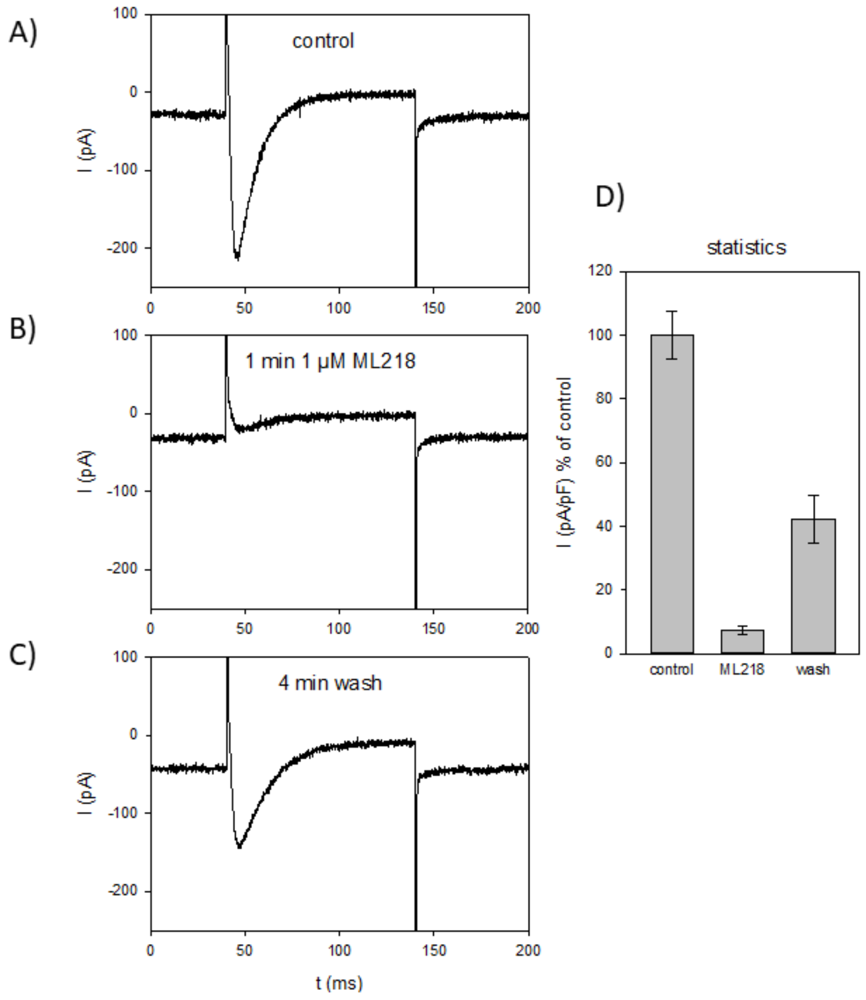

Effect of the T-type calcium channel inhibitor ML218 on $I_{Ca,T}$

Characteristic original current traces induced by depolarization from a holding potential of -90 mV to a test potential of -20 mV recorded under control condition (A), after treatment of HL-1-cells for 1 min with 1 µM ML218 (B) and after a 4 min wash out phase. The statistical evaluation of the peak current density of $I_{Ca,T}$ is shown in (D). The means ± SEM of 10 cells are significantly different ($p < 0.05$).

Suppl. Fig. 2

A)

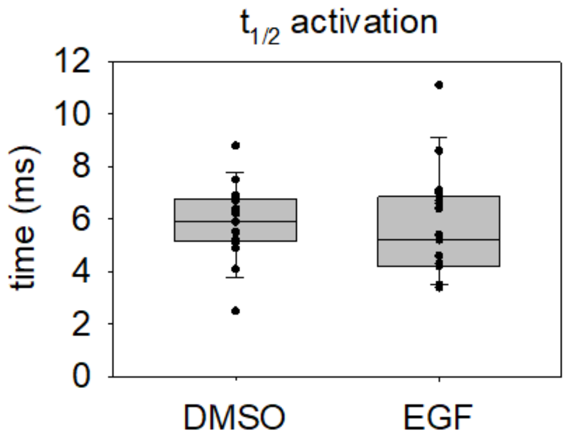

B)

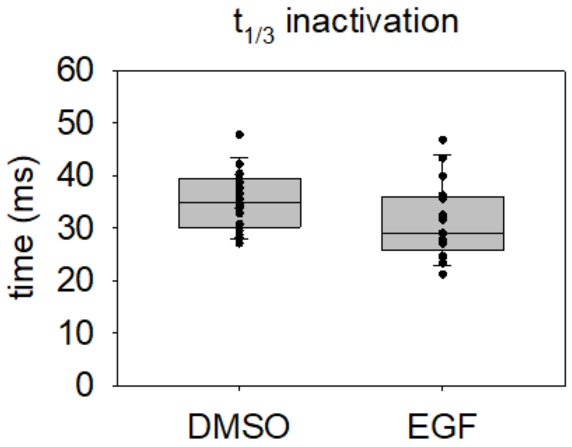

EGF does not affect the kinetics of T-type calcium channels

Time to half maximal activation ($t_{1/2}$ activation) (A) and time to 33 % inactivation ($t_{1/3}$ inactivation) (B) of $I_{Ca,T}$ were measured during or after, respectively, depolarizing the cells to a test potential of -30 mV starting from a holding potential of -90 mV . The means ± SEM of 17 cells are not statistically different.

Suppl. Fig. 3

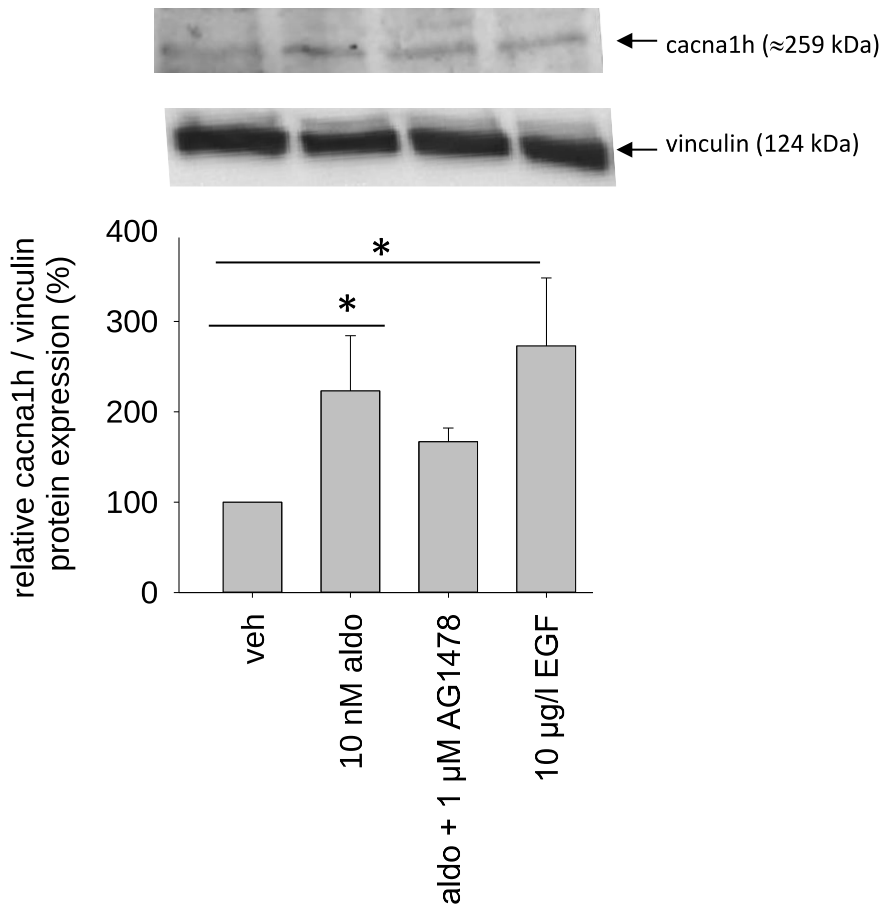

Effect of aldosterone, EGF and AG1478 on protein expression of Cacna1h in neonatal rat cardiomyocytes

Representative Western blot and quantification of protein expression of Cacna1h of rat neonatal cardiomyocytes incubated with veh (DMSO), 10 nM aldo, 10 µg/l EGF or 10 nM aldo and 1 µM AG1478 for 72 h (N=3; n=3; * $p \leq 0.05$).

Suppl. Fig. 4

Oligo (EMSA)	Sequence
MRE1.3sense	5'-Biotin-CGC CTG GTC CCT CCT CCT CCC GCC CTG CCT CCC CGC GCC TCG GCC CGC GC-3´
MRE1.3anti	5'-Biotin-CTC GGG ACT CCG GCC GCC TCG GCC GCG GCG GGC GCT CAC ACC GTG CGG GG-3´
MRE1.3-SNP1s<br>MRE1.3-SNP2s	5'-Biotin-GCA GCA GCC TCC GCC CCC CGC ACG G-3'
MRE1.3-SNP1a	5'-Biotin-CTC GGG ACT CCG GCC GCC TCG GCC GCG T-3'
MRE1.3-SNP2a	5'-Biotin-CTC GGG ACT CCG GCC GCC TCG GCC GCG G-3'

Sequences of the biotinylated probes used for EMSAs.

Suppl. Fig. 5

Oligo (qPCR)	Sequence
Accn1 sense	5'-GAGTGCCGATCCTCAGAGA-3'
Accn1 anti	5'-TGTAGCGGGTTAGGTTGCAG-3'
Cacna1c sense	5'-CCTGCTGGTGGTTAGCGTG-3'
Cacna1c anti	5'-TCTGCCTCCGTCTGTTTAGAA-3'
Cacna1g sense	5'-TGTCTCCGCACGGTCTGTAA-3'
Cacna1g anti	5'-AGATACCCAAAGCGACCATCTT-3'
Cacna1h sense	5'-CGTGACACTGGGCATGTTC-3'
Cacna1h anti	5'-TCCGTCCAGAGAGTATTCCATC-3'
Cacna1i sense	5'-GGGCGTGGCCTGTTTAGTC-3'
Cacna1i anti	5'-TGAGGGTCTCGGAGTGCTC-3'
Trpv2 sense	5'-TGCTGAGGTGAACAAAGGAAAG-3'
Trpv2 anti	5'-TCAAACCGATTTGGGTCCTGT-3'
Trpv4 sense	5'-CCTTGTTCGACTACGGCACTT-3'
Trpv4 anti	5'-GGATGGGCCGATTGAAGACTT -3'
Trpm7 sense	5'-AGGATGTCAGATTTGTCAGCAAC-3'
Trpm7 anti	5'-CCTGGTTAAAGTGTTCACCCAA-3'
Kcnn2 sense	5'-ACCACGCCAGGGAAATACAG-3'
Kcnn2 anti	5'-GGGCTGTCCATGTGAACGTA-3'
Clnc3 sense	5'-ACTCATACAACCCTGGCTGC-3'
Clnc3 anti	5'-CTGTGCAAAACACACCCGAG-3'
Clnc7 sense	5'-CTTCGTACTCGTGGGGTCTG-3'
Clnc7 anti	5'-GCTCCAGCTGAGACAAAATCC-3'

Sequence of primers used for detection of ion channel expression.

Suppl. Fig. S6

BNPsense	5’-CCAGTCTCCAGAGCAATTCAA-3’
BNPanti	5’-AGCTGTCTCTGGGCCATTTC-3’

Sequence of primers used for hypertrophy marker

Suppl. Fig. S7

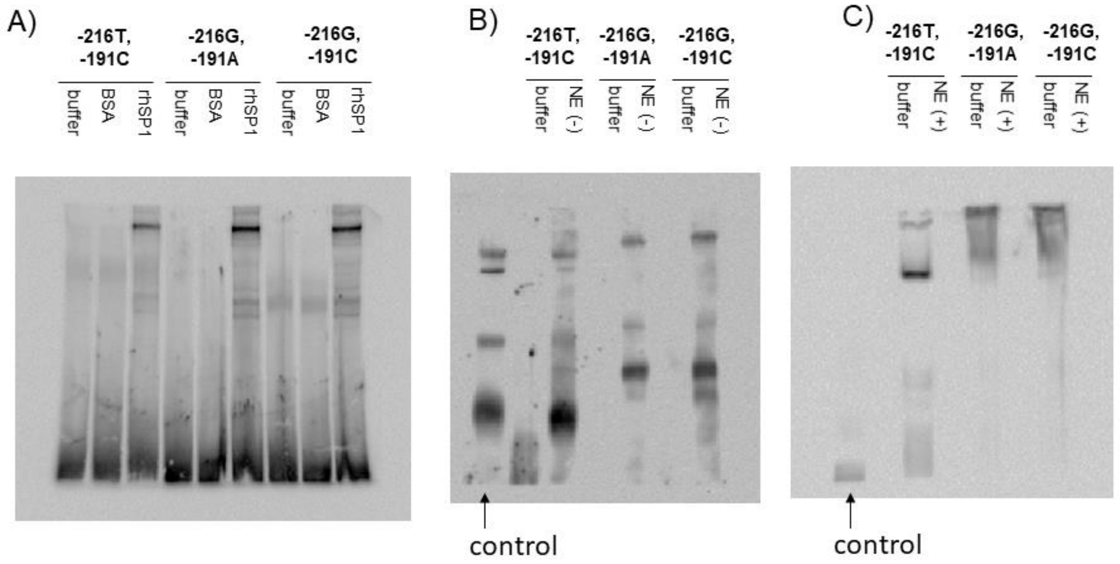

Detailed view of EMSAs shown in Fig. 1.

Suppl. Fig. S8

A)

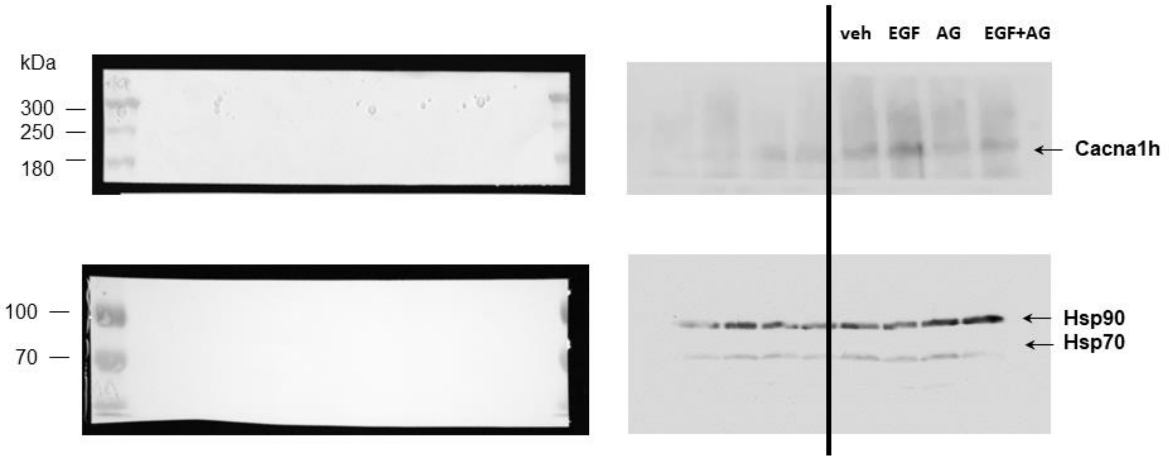

B)

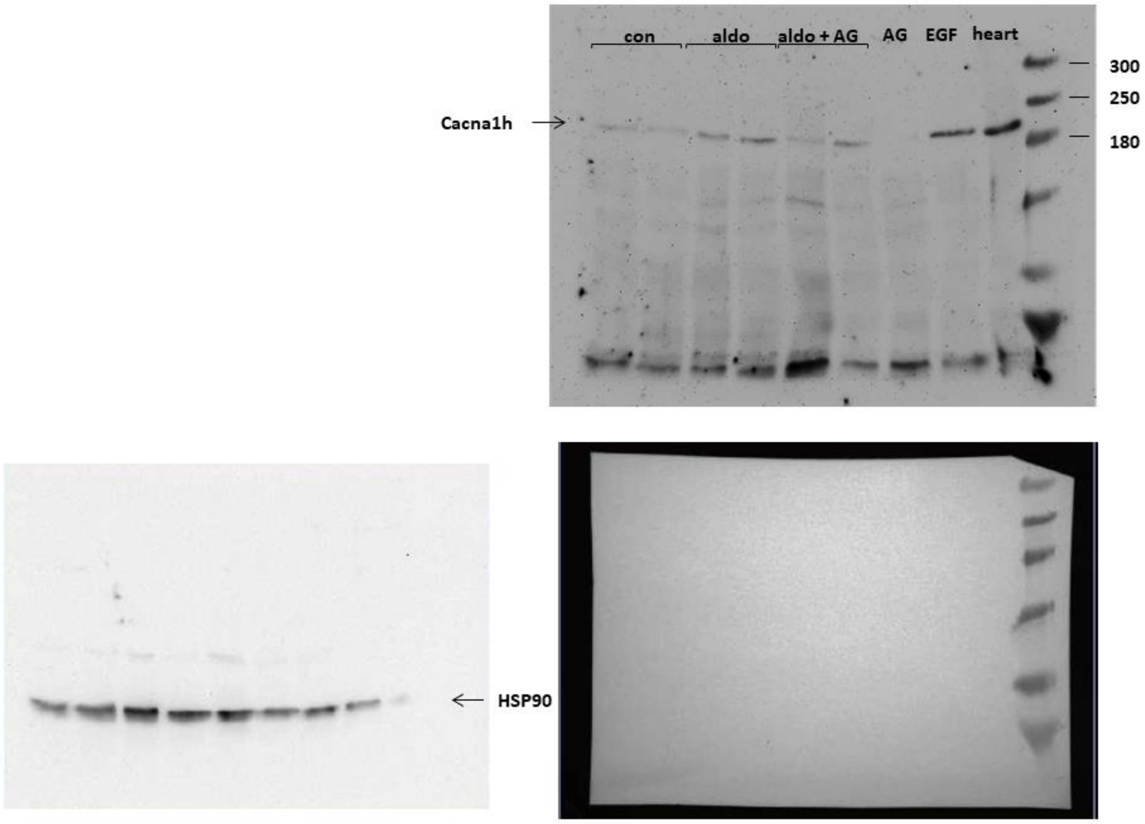

Detailed view of representative Western blots shown in Fig. 5D (A), H (B)(veh/con = DMSO, aldo = aldosterone, EGF = epidermal growth factor, AG = AG1478, heart = murine heart tissue as positive control).
